# Current and Future Applications of Toxicogenomics: Results Summary of a Survey from the HESI Genomics State of Science Subcommittee

**DOI:** 10.1289/ehp.0901501

**Published:** 2010-01-25

**Authors:** Syril Pettit, Shelley Ann des Etages, Louis Mylecraine, Ronald Snyder, Jennifer Fostel, Robert T. Dunn, Kenneth Haymes, Manuel Duval, James Stevens, Cynthia Afshari, Alison Vickers

**Affiliations:** 1 ILSI Health and Environmental Sciences Institute, Washington, DC, USA; 2 Pfizer Inc., Groton, Connecticut, USA; 3 Bayer HealthCare Pharmaceuticals, Montville, New Jersey, USA; 4 Merck & Co., Inc., Lafayette, New Jersey, USA; 5 SRA International, Inc., Durham, North Carolina, USA; 6 Amgen Inc., Thousand Oaks, California, USA; 7 U.S. Environmental Protection Agency, Washington, DC, USA; 8 Lilly Research Laboratories, Indianapolis, Indiana, USA; 9 Allergan Inc., Irvine, California, USA

**Keywords:** applications, HESI survey, impact and hurdles, toxicogenomics

## Abstract

**Background:**

In spite of the application of toxicogenomic (TGx) data to the field of toxicology for the past 10 years, the broad implementation and full impact of TGx for chemical and drug evaluation to improve decision making within organizations and by policy makers has not been achieved.

**Objectives:**

The goal of the Health and Environmental Sciences Institute (HESI) Committee on the Application of Genomics to Mechanism-based Risk Assessment was to construct and summarize a multisector survey, addressing key issues and perspectives on the current and future practical uses and challenges of implementing TGx data to facilitate discussions for decision making within organizations and by policy makers.

**Methods:**

An online survey to probe the current status and future challenges facing the field of TGx for drug and chemical evaluation in experimental and nonclinical models was taken by scientists and scientific decision/policy makers actively engaged in the field of TGx within industrial, academic, and regulatory sectors of the United States, Europe, and Japan. For this survey, TGx refers specifically to the analysis of gene expression responses to evaluate xenobiotic exposure in experimental and preclinical models.

**Results:**

The survey results are summarized from questions covering broad areas including technology used, organizational capacity and resource allocation, experimental approaches, data storage and exchange, perceptions of benefits and hurdles, and future expectations.

**Conclusions:**

The survey findings provide valuable information on the current state of the science of TGx applications and identify key areas in which TGx will have an impact as well as the key hurdles in applying TGx data to address issues. The findings serve as a public resource to facilitate discussions on the focus of future TGx efforts to ensure that a maximal benefit can be obtained from toxicogenomic studies.

The current state and future needs for application of toxicogenomic (TGx) technologies, including microarray analysis, to risk assessment was summarized recently in a National Academy of Sciences (NAS) publication [National Research Council (NRC) 2007]. The report points to the strong potential of the technology but acknowledges that neither the full impact nor broad implementation in the field of toxicology have been achieved. The NAS report identifies some of the practical challenges associated with array implementation, (e.g., cost, data storage requirements). The impact of genomics is palpable, especially in fields of gene and biomarker discovery, with succinct gene sets being correlated with human disease as well as response to treatment or stratification (Chanrion et al. 2007; Dressman et al. 2007; Rimkus et al. 2008). However, the impact to other specialized areas of science including toxicology is still being assessed (Foster et al. 2007; Gant 2007).

In this article we summarize a survey that gathered insights into the value and impact of TGx microarray gene-expression responses to evaluate chemicals and drugs in experimental and preclinical models. This survey, based on feedback from various user groups, offers unique insights complementary to the NAS report (NRC 2007). The survey was designed, written, and distributed by the Health and Environmental Sciences Institute (HESI) Committee on the Application of Genomics to Mechanism-based Risk Assessment. The goal of the survey was to obtain information on the current applications of TGx data and to identify the future challenges facing the field of TGx from different sectors (e.g., government, industry, academic) and job types (research vs. management) across the United States, Europe, and Japan. The survey was also designed to determine how various organizations are using microarray technology to evaluate the safety of chemicals and drugs regarding public health and regulatory assessment, the types of applications both *in vivo* and *in vitro*, the resources required, and the data-management challenges and solutions.

The HESI Genomics Committee is part of the nonprofit International Life Sciences Institute (ILSI) Health and Environmental Sciences Institute, a collaborative research organization that involves government, academic, and industrial scientists in the joint identification and resolution of toxicology and risk assessment issues.

The data generated by this survey provide a benchmark for the state of the science regarding applications of TGx data and identify the challenges concerning the implementation and impact of TGx data from experimental and preclinical models for the purpose of contributing to the safety evaluation of drugs and chemicals. The survey findings are intended to leverage experience about the value of TGx for chemical and drug evaluation regarding public health and to provide a frame of reference and resource to facilitate discussions within the scientific community and within organizations and by policy makers as the field of TGx continues to advance.

## Methods

### Survey design and analysis

The survey questions and format were collaboratively designed, written, and reviewed by multisector representatives from the HESI Genomics Committee. The final survey contained 62 multiple-choice questions focused on broad issue areas such as technologies employed, experimental approaches, organizational capacity and resource allocation, perceptions of benefits and hurdles, data storage and exchange, and future expectations. The survey was accessed and completed by respondents via the online survey hosting tool SurveyMonkey.com (http://www.surveymonkey.com/). Results were compiled by SurveyMonkey.com and then downloaded to a structured query language database for analysis.

### Survey distribution and response

An explanatory e-mail and link to the online survey was sent to approximately 300 potential respondents. The distribution list included scientists in the government, academic, and industrial (chemical, consumer products, and pharmaceutical) sectors and was populated from existing HESI mailing lists and contacts related to the HESI Genomics Committee. Our focus was to obtain the opinion and perspective of scientists and decision makers who are either directly involved in the field of TGx or review TGx data. We believed that individuals familiar with the field could provide valuable input on the status of the field and the potential hurdles, challenges, and future directions. For questions where the total number of respondents from a given sector was < 5, the data were excluded from the discussion as providing insufficient information.

A total of 112 respondents completed the survey (~ 30% response rate). The geographical distribution of respondents by site was approximately 64% from the United States, 25% from Europe, 6% from Japan, and the balance from other regions. Survey respondents were employed in a variety of sectors. The pharmaceutical industry (43%) accounted for almost half the respondents; government regulatory (26%) and research (11%) units accounted for another third; and academia (8%), biotechnology (7%), and chemical (< 5%) accounted for < 20% of participants.

About 70% of the respondents had experience using microarrays or worked in an organization that used the technology. The remaining 30% were not direct practitioners but used microarray data derived from external genomics-based studies. Job functions varied among respondents. Almost half described their job function as researchers or as managers in laboratory-based functions. Other job descriptions included executives, regulators, bioinformaticians, and some nonspecified roles (Table 1). Most of the respondents were aware of the HESI Committee on Genomics (90%) and believe that TGx will have a positive impact in preclinical safety assessment of drugs and chemicals.

## Results

The survey included a range of questions regarding toxicogenomic technologies used, organizational capacity and resource allocation within organizations, experimental application, data storage and exchange, and the impact and hurdles of TGx. The survey questions and results summary are available online (ILSI Health and Environmental Sciences Institute 2009).

### Microarray technology implementation

#### Technology resources

Survey respondents provided information on the types of genomics array technology used during the past 3 years (Table 2). Use was split evenly between one-channel oligonucleotide arrays and either two-channel or cDNA (complementary DNA) arrays with no particular sector-specific preferences observed.

#### Organizational capacity and resource allocation

The types of expertise retained in TGx groups varied, with no predominant must-have expertise. Approximately equal numbers of respondents across all sectors indicated that their groups retained some expertise in microarray and real-time polymerase chain reaction (PCR) technology, bioinformatics, and statistics, as well as biologic validation and follow-up activities. Expertise in regulatory framework applications was rarely reported as part of an internal TGx group, with the obvious exception of government regulators, who allocated up to 30% of internal staff for this activity.

Of the sectors surveyed, the pharmaceutical industry reported the highest total percentages of their full-time employees (FTEs) dedicated to laboratory work and data generation for TGx (~ 50% of staff dedicated to data generation). Government research and regulatory groups reported the lowest rates of in-house TGx data generation by FTEs, as most of their activities are conducted via partnerships or contract research. About 15% of academic and pharmaceutical sector respondents also reported outsourced research.

Across sectors, design of TGx experiments was almost exclusively accomplished internally except in those cases where the respondent was responsible for managing a research program (presumably because external collaborations, grants, and so on were involved). Probe generation and hybridization was also generally conducted internally, but external resources were required about 15–30% of the time across all sectors (academic, biotechnology, government, and pharmaceuticals), suggesting that this is not a core activity in some laboratories. Sample preparation was conducted externally about 15% of the time, possibly when the experimental work was outsourced or part of a collaboration effort, as indicated by responses from the academic, government regulatory, and government research sectors.

### Experimental applications of TGx

#### *In vitro* application of TGx

Survey respondents indicated that *in vitro* applications account for approximately half of their TGx efforts. The *in vitro* studies were based on work in cell lines (86%) and primary cultures (84%), as opposed to organ cultures (30%). Human, rat, and mouse were the most frequently used species and liver (82%) the most commonly used tissue. *In vitro* studies were used to identify mechanism of action (86%), compared with *in vivo* data (66%), as well as to identify potential biomarkers (61%) and compare animal and human tissue (52%).

#### *In vivo* applications of TGx

Respondents reported that their *in vivo* efforts generally focused on short-term studies (1–2 weeks) from rat, mouse, and dog, with the liver dominating as the most routinely processed tissue, followed by kidney. The other tissues processed sometimes included intestine, heart, peripheral blood leukocytes, skeletal muscle, lung, uterus, and brain. Some respondents reported the processing of whole blood for identification or measurement of biomarkers of toxicological response. Histopathology and clinical chemistry measures were cited as necessary anchors to place genomic findings into the appropriate biological context. Additionally, TGx data were viewed as important to evaluate 68% of the respondents. Integration of TGx data with other preclinical data, a systems biology approach to analyzing and interpreting the data, is used by 38% of the respondents, and another 34% consider using it in the future.

#### Use of non–array-based gene expression analyses

Respondents were asked about the use of non–array-based approach (e.g., real-time PCR) to assess expression levels of specific genes. Non–array-based analyses were used primarily to confirm array results (60%) as well as to confirm the identification of safety biomarkers (45%) and efficacy biomarkers (25%). Alternative measures of gene expression were also cited as a means to make repeated measures on a small number of transcripts (18%) and target characterization (23%). When asked how frequently confirmatory analyses support the primary microarray findings, there was a difference in the expected percentage of confirmation reported by laboratory directors (50% confirmation rate) and bench scientists (30% confirmation rate) compared with the responses from vice presidents (10% confirmation rate). These data suggest that the reliability of TGx technology is currently underestimated or incorrectly communicated at higher levels within organizational structure. When microarray results are followed by a second assay to detect gene expression, for example, real-time PCR, respondents from most sectors indicated that 60–89% of the microarray data are confirmed.

### TGx data storage and analysis

Although one of the great strengths of genomic microarrays is their ability to provide information about hundreds to thousands of gene sequences in a single assay, the storage and analysis of this information can pose significant challenges. This was highlighted in the survey by a series of questions relating to both the statistical approaches and database tools used to organize and interpret these large data sets.

Those using TGx technologies rely on a variety of resources to provide biological and mechanistic context to the gene changes observed via the microarray experiment. For example, commercially and publicly available pathway annotation, Gene Ontology (http://www.geneontology.org/) annotation, and Entrez Gene (http://www.ncbi.nlm.nih.gov/sites/entrez?db=gene) resources were used consistently across the sectors that were surveyed. These resources allowed users to access commonly referenced descriptions of gene products and their associated biological, cellular, and molecular functions. Database usage for data analysis by respondents was fairly evenly split between the internally developed and maintained databases versus publicly available databases. Not surprisingly, a higher percentage of academic and government scientists relied on public databases than did the private sector, which employed relatively more commercial in-house database products. When asked about the use of the public database resources, the same pattern followed; for example, government/academic scientists generally cited them as very or somewhat useful, in contrast to private sector respondents who were more equivocal in their praise for these resources.

Despite the complex bioinformatics expertise required to manipulate and interpret these massive data sets, only about half the respondents always or frequently consulted with a statistician when designing and analyzing a microarray study. Principal component analysis (PCA), hierarchical clustering, and analysis of variance/statistical analysis of microarrays (SAM) were the most commonly used statistical methods for analyzing microarray data across all the respondents.

Respondents considered data analysis and interpretation an important internal supporting expertise. The percentage of FTEs allocated to this activity varied widely, possibly a reflection of the mix and complexities of ongoing activities within each group. In most sectors, < 30% of FTEs were allocated for data analysis and interpretation, although some respondents from the pharmaceutical sector (8%), government research sector (25%), and biotechnology sector (40%) reported that 50–75% of available FTEs were allocated for analysis and interpretation.

Over the last several years, a number of standards for data formatting and data inclusion for TGx/microarray data have been developed. These include the MIAME (Minimum Information about a Microarray Experiment) conventions for reporting microarray data, the CEBS (Chemical Effects in Biological Systems) database standards, and the developing SEND (Standard for Exchange of Nonclinical Data) standards for reporting preclinical safety data. Almost half of the respondents comply with MIAME or MIAME-Tox standards in formatting their data per requirements put in place by many leading peer-reviewed journals. Significantly fewer (11%) cited the use of the SEND format.

Most of the respondents cited the value of sharing this type of data with collaborators (83%), with regulatory agencies voluntarily (67%), and with public databases (61%). Respondents identified the following as the greatest benefits of TGx data sharing: identification and consensus-building around novel biomarkers, standardization and harmonization of approaches and interpretation, and providing a means to inform the regulatory community about the application of TGx data.

### Impact of TGx

The greatest impact of TGx data viewed across sectors and job descriptions was the contribution to *a*) understanding biological mechanisms, *b*) identification of biomarker candidates, and *c*) identifying species differences.

**Figure 1:**
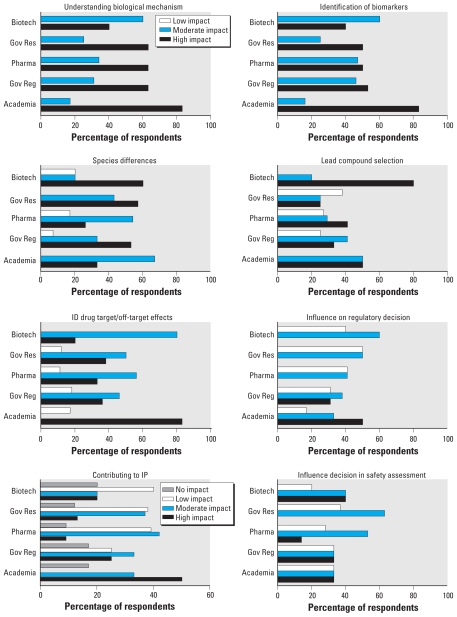
Major reasons cited as to the impact of TGx data from the sectors of biotechnology (Biotech), government research (Gov Res), pharmaceutical companies (Pharma), government regulatory agencies (Gov Reg), and academia. Chemical industry responses were < 5% and are not shown.

These areas were regarded as the most relevant applications for TGx. The ability to influence lead compound selection was viewed as moderate to high impact, whereas identifying drug target and off-target effects were viewed as having a moderate impact. The use of TGx as a supplemental or supportive data source for decision making was cited by about half of the respondents as having a moderate impact. Respondents from academia regarded this to be a high-impact area for TGx, whereas the pharmaceutical sector responded that the impact would be low. The government regulatory respondents were more positive that TGx data could be supportive (31% high, 38% moderate, and 31% low). The contribution of TGx to intellectual property was regarded as having a moderate impact (Figure 1).

### Hurdles of TGx

**Figure 2:**
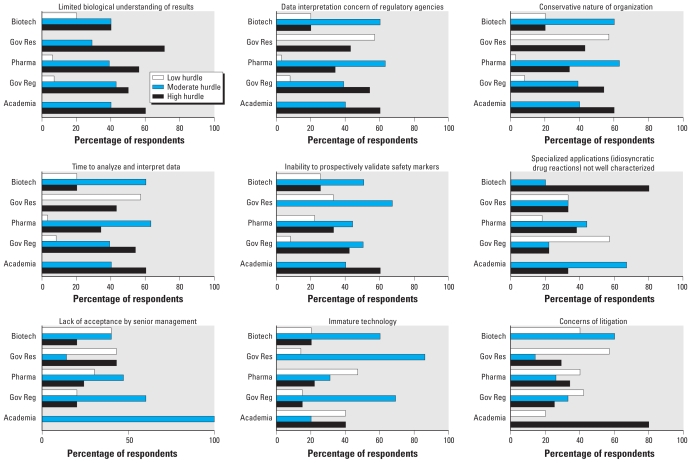
Major reasons identified as to the hurdles of implementing TGx data from the sectors of biotechnology (Biotech), government research (Gov Res), pharmaceutical companies (Pharma), government regulatory agencies (Gov Reg), and academia. Chemical industry responses were < 5% and are not shown.

As a reflection of the diversity of groups that conduct TGx studies, there appears to be no single driving factor that currently limits application of TGx technology (Figure 2).

Even though TGx is contributing to understanding biological mechanisms, it is still the opinion of virtually all sectors that the current limited biological understanding of TGx data remains as a high hurdle to increasing the impact (≥ 50% respondents for each sector) of TGx data. Interpretation of TGx data by regulatory agencies was considered to be a hurdle of high concern by government regulatory respondents (54%) and academia (60%). Most job sectors viewed this hurdle as a moderate to high concern. A conservative nature of organizations was also seen as a moderate to high concern by most sectors. If understanding of biological context is important for mechanisms, the survey may indicate that additional information remains to be gained for TGx data to have a greater impact.

Additionally, across sectors, about 20% of respondents continue to identify insufficient maturation of the technology and concerns about a lack of return on investment as limitations. Other hurdles considered moderate included time to analyze and interpret data; inability to prospectively validate safety markers; specialized applications, such as identifying idiosyncratic drug reactions; lack of acceptance of approach by senior management within companies; and concerns of litigation from retrospective analysis.

Challenges associated with the exchange and formatting of data were cited as being of higher concern by public sector regulatory respondents than by other respondents. This response is consistent with publicly available guidance documents and presentations [Food and Drug Administration (FDA) 2007], indicating that many regulatory agencies are still developing policies and database/data storage resources to address the receipt and storage of large data sets from a variety of sources.

Those sectors less heavily invested in the use of TGx for basic research also cited lack of staffing and analytical capabilities as an impediment to broader implementation. In the academic and government sectors, insufficient budgets for equipment, supplies/reagents, and outsourcing are perceived as limiting factors by up to approximately 25% of responses.

### TGx in safety and risk assessment

#### Role in regulatory submissions

Ultimately, one of the most important benchmarks regarding the impact of TGx technology in safety assessment will be the inclusion of the data in regulatory submissions. Respondents were asked to answer questions regarding the current and future impact of TGx data on regulatory submissions.

At present, there are no requirements for submission of preclinical TGx data to major regulatory agencies in the United States, Europe, or Japan. However, most of these agencies have produced draft guidance documents and are encouraging submission on a voluntary basis:

- *Guidance for Industry: Pharmacogenomic Data Submissions* (FDA 2007)
- *Genomic Data Submission* (FDA 2009)
- *External Review Draft of the Interim Guidance for Microarray*-*Based Assays: Data Submission, Quality, Analysis, Management and Training Considerations* (U.S. Environmental Protection Agency 2009)
- *Scientific Guidelines for Human Medicinal Products* (European Medicines Agency 2009).

Respondents from regulated industries (17%) had submitted voluntary data, but nearly 50% suggested they would consider submission in the future. About 20% of pharmaceutical respondent organizations had participated in a submission, but > 50% of the executive respondents from this sector felt the practice will increase in the future.

However, most of the respondents from the government regulatory sector felt that submissions would be useful and would significantly improve the safety assessment process by helping to explain mechanisms of toxicity. About half of all the regulators also expressed some concern about the lack of resources/approaches for analyzing these data within their agencies. Most of the respondents from government regulatory (85%) and research (75%) sectors also felt that it would be beneficial to have a requirement to submit microarray data, whereas pharmaceutical (53%) and academic (50%) respondents were less convinced. Although more than half of the respondents felt that there was value in providing TGx data as part of voluntary submissions to regulatory agencies, only about one-third cited value in sharing these data as part of a required regulatory submission.

In contrast to the apparent enthusiasm to share microarray data, substantial barriers may prevent these data from being shared. Almost 80% of the respondents cited intellectual property/patent concerns as a potential barrier to the sharing of TGx data, and nearly 50% of respondents also cited concerns about variability in data formats, interplatform differences, concerns about potential for misinterpretation, lack of incentive to share data, and legal concerns.

The main benefits of submitting TGx data as part of a submission package cited by respondents included the following:

- Supporting the molecular basis of toxicity in response to a drug, or to chemicals in food and in the environment (80%)
- Aiding in the acceptance of biomarkers (50%)
- Contributing significantly to an improved safety assessment of products (50%)
- Providing a stronger regulatory submission package (40%)
- Identifying susceptible populations (40%).

However, respondents also expressed concerns that submission of TGx data as part of a submission package might not be beneficial. Their concerns included the following:

- Some of the regulatory agencies have not defined methodology to analyze genomic data in the existing safety assessment paradigm (45%).
- Until the molecular mechanisms and pathways of toxicity are better understood, genomic data will be of limited use in the safety assessment paradigm (43%).
- Without established methods and a better understanding of mechanism, regulatory agencies may interpret the microarray TGx data differently and come to a different conclusion (43%).

When questioned about the role of TGx data in influencing regulatory decisions, most private sector respondents indicated that the anticipated impacts over the next 2–5 years would be moderate to low. Government regulators responding to the survey predicted a moderate to high impact in this time frame (31% high, 38% moderate). It was not possible to discern from the survey responses if this moderate level of enthusiasm represented the current state that could improve in the future or a general moderation in the overall enthusiasm for the potential for TGx to alter the science of toxicology.

### Future uses of TGx

There was broad consensus across job sectors (industry, academia, and government) as well as scientific positions (research and applied scientists, management, and policy makers) that toxicogenomic data would have a moderate to high impact in a variety of areas of safety and risk assessment over the next 2–5 years. Among the areas for which consensus emerged regarding the potential impact by TGx in the near future are *a*) understanding the biologic mechanism of action of the toxic effect, and *b*) identification of candidate biomarkers of toxicity.

Additional areas in which the respondents considered that TGx will contribute knowledge included influencing lead compound selection with respect to compound safety potential, identifying species differences in toxicity, and identifying drug target and off-target effects. Respondents were less optimistic about TGx data influencing the decision-making process in safety assessment, influencing regulatory decisions, and contributing to intellectual property for products or for a class of products.

## Discussion

There is little doubt that genomics-based tools and analytical techniques are beginning to pay dividends to scientists and organizations who dedicate time and resources to these tools. One metric is the number of publications that derive some or all of their content from genomics-based methods. The past 7–10 years have exhibited a sharp rise in the number of scholarly articles relying on data obtained using genomic techniques. Less obvious, perhaps, are the current hurdles facing the TGx field and clear direction of the field in the immediate future. The present survey of a broad user base of TGx technologies was intended to both assess the state of the science today and identify real and potential barriers to progress. The data not only provide valuable information on the current state-of-the-art but, more important, also serve as a starting point for focusing future efforts to ensure that maximal benefit can be obtained from TGx studies.

From a demographic perspective, the survey respondents represented a cross-section of industry, government, and academic researchers from a variety of roles. Geographically, respondents were primarily from Europe, Japan, and the United States, which is not surprising given that most of the academic and industrial genomics data are produced from these regions. About half of the respondents identified themselves as in-laboratory scientists or as managers of a laboratory. Thus, survey data related to technical aspects of genomics should be accurately represented. Of particular interest is the responses of the executives who allocate resources versus the technical practitioners of the science with regard to TGx and how the science can be managed as the field advances.

One clear and important point that emerged from these survey data was that there is a disconnect on the perceived value of TGx data from the viewpoint of the scientists who generate the data and the people in organizations who have to defend the dedication of resources to these expensive technologies. For example, executives felt that confirmatory assays rarely led to a true confirmation of an initial result from a genomics experiment, whereas scientists more often felt that a secondary assay did indeed provide confirmation. The important question is whether the source of this apparent contradiction is a true disagreement between data sets or, perhaps more seriously, a failure to correctly communicate these data. One thing is certain: The future of genomics technology is absolutely dependent on both data quality and quality of interpretation. Thus, accuracy and perception of data are both key areas for focus in the coming years. It certainly bodes well for both accurate interpretation and communication of genomics data that computational tools and techniques are becoming more mainstream and user friendly. However, the availability of qualified bioinformatics experts and individuals who can effectively integrate the genomic/computational output with toxicological interpretation is still a crucial need for the field—one that will probably lag behind the technology until training programs for bioinformatics toxicology specialists reach critical mass.

Another surprising result from the survey was the report of use of samples derived from *in vitro* studies for genomics analysis. The use of cell lines (86%) equaled the use of primary cultures (84%), followed by the use of organ cultures (30%). These data are intriguing, given that *in vitro* models are often criticized for lack of relevance to more complicated *in vivo* systems, especially in toxicology studies where risk assessments are often based on highly integrated models. There are a number of reasons why *in vitro* models may be favored for use in genomics studies. First, because of their inherent simplicity, data interpretation can be more straightforward. This assertion is true when the research questions focus on receptor or pathway activation or inhibition. The downstream mediators for most receptor systems, either membrane-bound or soluble, are fairly well understood. For example, the battery of genes involved in signaling in response to aryl hydrocarbon receptor activation are very well characterized in a number of cell lines (Rivera et al. 2007). This suggests that genomics can provide rapid insight into pathways potentially involved in mechanisms of action or toxicity or into discovery of new ligands or inhibitors. However, more complex questions such as those involving bioactivation or complex metabolic cascades are still probably best addressed in more completely integrated physiological systems. Second, the cost of generating samples from *in vitro* systems is substantially less than the costs required to generate samples from *in vivo* systems, even when one considers the expense of the infrastructure required to maintain *in vivo* systems. The cost factor is especially important, given the fairly high cost of microarray platforms and the subsequent burden the resulting data place on experts in an organization in terms of time and electronic data management. Thus, running inexpensive experiments to generate samples for use on an expensive platform makes intuitive sense as long as the science is sound. Finally, *in vitro* systems offer the advantage of rapid screening using smaller amounts of material such as compounds for pharmaceutical screening. It may be possible to explore a wider chemical space in a shorter time using *in vitro* systems. Although precise reasons for leveraging use of *in vitro* models for generation of genomics data are perhaps elusive, the practical aspects cited above for use of *in vitro* systems undoubtedly play a role.

This survey also included a series of questions intended to characterize the challenges of genomic data management, use, and sharing. The survey demonstrated the perception that there is value in sharing genomics data both within an organization and between organizations. However, potential concerns did emerge with regard to data sharing. The primary issue cited in these concerns centered on intellectual property (i.e., patent and freedom to operate issues). The relatively large contribution of survey responses from private industry may have added some bias to the issue of proprietary concerns, but these concerns are nonetheless valid and important as the field of genomics continues to mature. It should be mentioned that academic institutions are also vested in intellectual property concerns, as most prominent universities have a very keen sense of value that can be derived from discoveries made by their own scientists and have resources in place to protect those discoveries.

It was not surprising that there was little consensus on the use of data standards for reporting TGx data. Other than the generally accepted MIAME convention, there is little standardization for reporting orthogonal data relevant to interpretation of microarray results (e.g., pathology results or biochemical assay data), nor are developing standards for reporting data from nonclinical safety studies widely accepted. The responses indicate that a lack of standardization will continue to represent a barrier to sharing microarray data and to the development of public databases. Thus, the lack of consensus on the use of data standards may reflect a lack of focus and understanding of an important and potentially rate-limiting step for acceptance of TGx data.

The disparity in the views regarding the expected impact that TGx data will have on regulatory decisions (i.e., high to medium by academics and regulators versus low by the private sector) suggests that additional dialog among stakeholders is essential. For example, if there are areas of clear need that can be supported by TGx data in the context of regulatory submissions, they need to be defined and articulated. On the other hand, if the technology is finding wider acceptance within the private sector in nonregulated areas, then there may be a misunderstanding with regard to the most likely uses of the technology. Regardless, understanding these differences in opinion will be critical to achieving good partnership between the public and private sectors in advancing the technology.

## Figures and Tables

**Table 1:** Survey respondents (*n* = 112) according to job position.

Position	Percentage of respondents
Laboratory researcher	25
Laboratory director	20
Research program manager	8
Informatics/database manager	8
VP/executive decision maker	9
Regulatory development	15
Other	15

VP, vice president.

**Table 2:** Type of high-density (> 6,000 data points) microarray technologies employed by respondents.

	Respondents using technology (%)		
Array type	In 2005	In 2006	In 2007
One-channel oligo microarray	38.2	40.2	45.1
Two-channel oligo microarray	22.5	22.5	21.6
cDNA microarray	19.6	17.6	16.7
Other high-density transcriptomics	6.9	6.9	11.8
